# A proteomic view of isoproterenol induced cardiac hypertrophy: Prohibitin identified as a potential biomarker in rats

**DOI:** 10.1186/1479-5876-11-130

**Published:** 2013-05-24

**Authors:** Debabrata Chowdhury, Anjana Devi Tangutur, Tarak Nath Khatua, Priyanka Saxena, Sanjay K Banerjee, Manika Pal Bhadra

**Affiliations:** 1 Centre for Chemical Biology, Indian Institute of Chemical Technology, Uppal Road, Hyderabad 500607, India; 2 Division of Medicinal Chemistry and Pharmacology, Indian Institute of Chemical Technology, Uppal Road, Hyderabad 500607, India; 3 Centre for Cellular and Molecular Biology, Uppal Road, Hyderabad 500607, India

**Keywords:** Cardiac hypertrophy, Isoproterenol, Proteome analysis, 2D-gels, Differentially expressed proteins, Prohibitin

## Abstract

**Background:**

The present study aimed at using a proteomics based approach to: a) analyze and contrast the proteome of the healthy and isoproterenol induced hypertrophied hearts and b) identify potential biomarkers for diagnosis of cardiac hypertrophy.

**Methods:**

Male Sprague Dawley (SD) rats were administered isoproterenol (ISO, 5 mg/kg, sc, once daily) for 14 days to induce cardiac hypertrophy. There was a significant (p<0.05) increase (~ 55%) in the heart weight to tail length ratio after 14 days of treatment and cardiac hypertrophy was evidenced by significant increase of β-MHC and ANP, two indicative markers of cardiac hypertrophy, in the treated heart compared to that of control. Following confirmation of hypertrophy, 2DE of the tissue samples was done followed by MS/MS analysis of the protein spots to obtain a proteomic view for identification of novel biomarkers.

**Results:**

Several important proteins were identified by proteomics analysis. They belong to the major functional categories such as cholesterol and protein metabolism, muscle contraction and development, transport, TCAcycle, ATP-biosynthesis, chaperone, signal transduction, DNA synthesis and ubiquitinisation. Careful examination of these protein spots by image analysis led to the successful identification of 7 differentially expressed proteins in the diseased sample. Further extension of this work for validation of differential expression of these proteins was also achieved by RTPCR and western blotting.

**Conclusions:**

Our results demonstrate characteristic protein expression profile in control and hypertrophy condition in SD rats and also expand the existing knowledge on differentially expressed proteins in hypertrophy. The study signifies the importance of reduced expression of a novel protein such as Prohibitin (PHB) which may be associated with the cardiomyocytes growth and cardiac hypertrophy. However, further work is necessary to confirm the role of PHB in human heart and its potential role in diagnostic and therapeutic intervention in the clinic.

## Background

Cardiac hypertrophy leading to heart failure is one of the major causes of morbidity and mortality in the world. Cardiac hypertrophy is characterized by a chronic physiological increase in cardiac muscle mass resulting from systolic or diastolic wall stress. This often occurs normally during development, during pregnancy, and in response to sustained exercise [1]. However, if this condition persists, although initially compensatory for an increased workload, prolongation and prevalence of this process leads to congestive heart failure, arrhythmia, and sudden death [2]. It may also result from a number of pathological conditions such as hypertension, valvular disease, myocardial infarction and cardiomyopathy [3]. At the cellular level, cardiac hypertrophy is characterized by an increase in cell size, protein synthesis and reactivation of the fetal gene program finally leading to heart failure [4].

Isoproterenol, a catecholamine, induced cardiac hypertrophy represents the most widely used model which mimics the sustained adrenergic stimulation and represents an important hallmark of the pathogenesis of maladaptive cardiac hypertrophy [5]. The activation of β-adrenergic signaling in turn induces in the heart many different mechanisms which contribute to the hypertrophic phenotype including enhanced protein synthesis, proto-oncogene expression, elevated oxidative stress, stimulation of mitogen activated protein kinases and phosphotidyl inositol-3 kinases [6]. Studies using transgenic mice with heart-specific overexpression of β1-adrenergic receptors had increased cardiac contractility at a young age and also developed marked myocyte hypertrophy. Cardiac hypertrophy was followed by progressive heart failure with functional deficits similar to human heart failure [7].

While studies that focus on specific genes or pathways have provided insights over the years in human and animal models [8], integrative approaches are equally important in identifying multiple pathways or novel markers that are involved in the disease progression and mechanisms. The changes in protein expression in the myocardium are critical elements for understanding the molecular mechanisms of myocardial remodeling or identifying useful biomarkers for diagnostic purpose and therapeutic intervention. Two-dimensional gel electrophoresis coupled with mass spectrometry offers a key advantage of profiling of thousands of proteins in the biological samples. We therefore aimed to investigate the alterations in the proteome profile of healthy and isoproterenol induced hypertrophy in the Sprague–Dawley rats by proteomics approach using two-dimensional gel electrophoresis in conjunction with matrix assisted laser desorption ionization - time of flight (MALDI-TOF) mass spectrometry to increase our understanding of the mechanistic pathways involved and to identify novel potential biomarkers for diagnosis and prognosis.

## Methods

### Animals and treatment

The experiments were initiated on 6–7 week old male Sprague Dawley (SD) rats weighing between 120 –160 g, obtained from National Institute of Nutrition (NIN), Hyderabad, India. The animals were housed in BIOSAFE, an animal quarantine facility of Indian Institute of Chemical Technology (IICT, Hyderabad, India), maintained at a temperature of 22 ± 2°C and relative humidity of 50±15%. Animals were acclimatized for a period of one week before the start of the study. A 12 hour dark/light cycle was maintained throughout the study. Air changes (15–16 cycles/hr) were maintained with 5 μ HEPA filter. Rats had free access to food (pellet diet supplied from National Institute of Nutrition, Hyderabad) and water *ad libitum.* The study protocols followed were in accordance with the guidelines of the Institutional Animal Ethics Committee of Indian Institute of Chemical Technology, Hyderabad and conform with the Guide for the Care and Use of Laboratory Animals published by the US National Institutes of Health (NIH Publication No. 85–23, revised 1985). For the development of hypertrophy all animals were weighed, and randomly divided into two groups: Control (Con: saline administration through subcutaneous route, 14 days, n=8) and Hypertrophy (Hyp: isoproterenol administration through subcutaneous route, 5 mg/kg/day, 14 days, n= 8) [9]. Food and water intake was recorded daily. Body weight was taken at the start and end of 14 days. After sacrificing the rats, the heart tissues were carefully isolated and excised. Then hearts were washed three times with 1X PBS (pH-7.4) and stored in −80°C freezer for future use.

### Estimation of heart weight/tail length ratio

In each group, heart weight/tail length ratio was measured on the day of sacrifice as a parameter of cardiac hypertrophy. Tail length was measured by using a centimeter (cm) scale. Heart weight (gm) was measured after keeping the heart in ice cold saline and blotting out the heart with tissue paper [10].

### β-MHC and ANP gene expression as markers of cardiac hypertrophy

Total RNA (DNAase free) was isolated from heart tissues using TRI REAGENT (Sigma, St. Louis, MO, USA). Reverse transcription was performed by using the MMLV reverse transcriptase kit (M6125H, Epicenter, WI, USA) for first-strand cDNA synthesis [11]. Real-time PCR was performed by using SYBR^®^ Green PCR Master Mix (2X) (Takara, Takara Bio. Inc., Japan) and following gene specific primers: forward primer, 5′-AGAGCAAAAGCAAAGGGTTTC-3′ and reverse primer, 5′-GTGATGGTACGAGATGGGCTA-3′ for β-MHC and forward primer, 5′- AGCGAGCAGACCGATGAAG-3′ and reverse primer, 5′-AGCCCTCAGTTTGCTTTTCA-3′ for ANP. PCR amplification of RPL32 (16S Ribosomal protein L32) cDNA was performed as reference control by using the following primer pair: forward primer, 5′-AGATTCAAGGGCCAGATCCT -3′ and reverse primer, 5′-CGATGGCTTTTCGGTTCTTA-3′*.*

### Protein extraction and quantitation

Approximately 80 mg of the heart tissue of control and cardiac hypertrophy samples were homogenized in a ratio of 1:20 with RIPA buffer in an electrical homogenizer followed by sonication with sonicator. The samples were then centrifuged at 14,000 rpm for 30 min at 4°C. The supernatant was collected and precipitated by standard TCA precipitation method. The pellet so obtained was solubilized in a sample buffer (8.0 M urea, 2% CHAPS, 50 mM DTT) at 4°C. The protein concentration of these protein extracts was determined using the Bradford protein assay kit (Biorad, Hercules, CA, USA) according to the manufacturer’s instructions using BSA as a standard. This protein sample could be used for 2-DE or stored in −70°C until used. Biolyte 3/10 ampholytes (0.2%) and bromophenol blue (trace) were added to the protein sample prior to 2-DE (rehydration).

### One dimensional gel electrophoresis (1-DE)

One dimensional SDS-PAGE was initially performed for preliminary proteomic profiling of control and hypertrophic heart samples according to Laemmli [12].

### Two dimensional gel electrophoresis (2-DE)

Protein samples (200 μg) were applied to an immobilized pH gradient (IPG) strip (7 cm, pH 4–7, Linear) (BioRad, Hercules, CA, USA) using a passive rehydration method (16 h of rehydration at 20°C) according to the manufacturer’s instructions. The IPG strips were then transferred to an isoelectric focusing (IEF) cell (BioRad, Hercules, CA, USA) and IEF was performed on a 7cm gel strip according to the manufacturer’s instructions. Prior to second dimension, the gel strips were equilibrated for 15 min twice in equilibration buffers I and II as previously described. The second dimension was performed using 12% SDS-PAGE at 80 V. The gels were stained using the fast-coomassie staining method and scanned with a BioRad GS-800scanner. At least two independent runs were performed for each sample to ensure the accuracy of analyses. The 2D gel maps were analysed by the ‘Progenesis same spots software’ (Non-linear dynamics, Durham, NC, USA). The quantity of each spot in a gel was normalized as a percentage of the total quantity of all spots in that gel and evaluated in terms of optical density (OD). Only spots that showed consistent and significant differences were selected for MS analysis.

### In-gel trypsin digestion

In-gel digestion of proteins was carried out using MS-grade Trypsin Gold (Promega, Madison, WI, USA) according to the manufacturer’s instructions. Spots were cut out of the gel (1–2 mm diameter) using capillaries, and destained twice with 25 mM NH_4_HCO_3_/50% acetonitrile (ACN) at room temperature for 45 min in each treatment. This was followed by dehydration of the gels with 100% ACN for 5 min. After dehydration and drying, the gels were pre-incubated in 10–20 μl trypsin solution (20 ng/μl) for 1 h. Then samples were added in adequate digestion buffer (25 mM NH_4_HCO_3_/50% ACN) to cover the gels and incubated overnight at 37°C. Tryptic digests (peptides) were extracted twice with 50% ACN/5% trifluoroacetic acid (TFA) for 30 min each time. The combined extracts were dried in a vacuum concentrator at room temperature. The extracted peptides were dissolved in 2.5 μl of 50% ACN/0.1% TFA, and then 0.8 μl of the digests was mixed with 0.8 μl of 5 mg/ml alpha-cyano-4-hydroxy-cinnamic acid (CHCA) (Applied Biosystems, Framingham, MA) in 50% ACN/0.1% TFA and spotted onto a MALDI target plate.

### Mass spectrometry (MALDI-TOF analysis)

The samples were analyzed on a 4800 Proteomics Analyzer MALDI-TOF/TOF mass spectrometer (Applied Biosystems, Framingham, MA) working in positive ion reflector mode. Peptide mass fingerprint (PMF) of the tryptic digests was acquired in an automation mode. MS/MS was performed in a data-dependent mode in which the top ten most abundant ions for each MS scan were selected for MS/MS analysis. The MS/MS data were acquired and processed using the global proteomics software and MASCOT was used to search the database. This software creates a peaklist from the raw spectra with a given threshold after removal of trypsin, polymer peaks, noise peaks and subjected to database interrogation for protein identification.

### Protein identification

PMF data was interrogated for protein identification with NCBI database for *Rattus* using the mascot search engine (http://www.matrixscience.com). Searches were performed without constraining protein molecular weight or isoelectric point, with complete carbamidomethylation of cysteine, partial oxidation of methionine residues, and 1 missed cleavage was also allowed in the search parameters. Proteins with probability-based Molecular Weight Search (MOWSE) scores exceeding their threshold (*P*<0.05), number of matched ions, number of matching ions with independent MS/MS matches, percent protein sequence coverage, and correlation of gel region with predicted MW and pI were collectively considered for each protein identification. The peptide and fragment mass tolerance were set at 100 ppm, respectively.

### RNA isolation and Reverse Transcriptase PCR (RTPCR)

Total RNA was isolated and Reverse transcriptase-polymerase chain reaction(s) were performed as described earlier in section 2.3. PCR was performed by using dNTPs Taq DNA polymerase, MgCl_2_ and specific primers according to the manufacturer’s instructions (Takara Taq kit, Takara Bio. Inc., Japan). The primers for RTPCR analysis (Table 1) were designed using Primer 3 software (from published sequence information), avoiding regions of homology with other genes, for the following genes: MYL-2, MYL-3, PEBP-1, PHB, HSP60, ATP5β and Desmin. The PCR product was separated on a 2% agarose gel stained with ethidiumbromide. The gel images were captured using gel documentation system and quantification of bands was performed using the Image J Software (NIH, Bethesda, MD, USA). For quantitative analysis, the signal intensity of each band was normalized with the GAPDH signals.

**Table 1:** List of primer sequences for gene of interest

**Gene of interest**	**Primers**	**Sequence**	**T**_**m**_
MYL2	Forward primer	5'-GAAAGCCAAGAAGAGGTTAGAGG-3'	55°C
	Reverse primer	5'-ATGGTGAGGAACACAGTGAAGTT-3'	
MYL3	Forward primer	5'-AAGAGCTCAACTCCAAGATGATG-3'	55°C
	Reverse primer	5'-TCTCTACCTCGTCTTCTGTCAGC-3'	
PEBP1	Forward primer	5'-GTCATGAATAGACCAAGCAGCAT-3'	57°C
	Reverse primer	5'-CTCATACACCAGCCAGACGTAG-3'	
PHB	Forward primer	5'-GGGTACAGAAGCCAATCATCTTT-3'	55°C
	Reverse primer	5'-AATGCTGGTGTAGATACGAGGAA-3'	
HSP60	Forward primer	5'-GCAGAGTTCCTCAGAGGTTGG-3'	53°C
	Reverse primer	5'-CCCAGCAGCATCCAGTAAAG-3'	
Atp5b	Forward primer	5'-TGTATTTGCTGGTGTTGGTGA-3'	53°C
	Reverse primer	5'-ACCTTGGAAGTGGCATCTTTT-3'	
Desmin	Forward primer	5'-CTGATAGACGACCTGCAGAGG-3'	55°C
	Reverse primer	5'-AAGGAATGCAATCTCCTCGTT-3'	
GAPDH	Forward primer	5'-TGGTGCTCAGTGTAGCCCAG-3'	58°C
	Reverse primer	5'-GGACCTGACCTGCCGTCTAG-3'	

### Immunoblot analysis

Total protein extraction and immunoblotting was performed as described previously [13]. Protein concentration was determined by Bradford method (Bio-Rad, Hercules, CA, USA). An equal amount (20 μg) of protein of each of the samples under study was separated by sodium dodecyl sulfate polyacrylamide gel electrophoresis (SDS–PAGE). After electrophoresis, protein was transferred to PVDF membranes (GE Healthcare, USA). The membranes were then blocked in Tris-buffered saline Tween-20 (TBS-T; 10 mM Tris, pH 7.5, 150 mM NaCl, 0.05% Tween-20) and 5% non-fat dry milk for 1 h, and subsequently washed and incubated with primary antibodies in TBST with 2.5% non-fat dry milk at 4°C for overnight. The following polyclonal antibodies and titres were used: MYL2 (1:20,000, LS BioScience #LSc105679), MYL3 (1 μg/ml, LS BioScience # LSc107502), PEBP1 (0.02 μg/ml, LS BioScience # LSb3247), PHB (2 μg/ml, LS BioScience # LSc29731), Hsp60 (1:300, Abbiotec #250700), ATPase 5β (1:500, LS BioScience # LS- C137091) and β-actin (1:1000, abcam #ab8224). After washing with TBS-T, membranes were incubated with Goat Anti-Rabbit IgG–HRP (1:1500 dilution, Santa Cruz Biotechnology, # SC 2004) or Goat Anti-Mouse IgG-HRP (1:1500 dilution, Santa Cruz, #2005) horseradish peroxidase conjugated secondary antibody with 2.5% non-fat dry milk at room temperature for 1 h. After washing with TBS-T immunoreactions were visualized with a chemiluminescence detection kit (Prod No- 34080, Super signal® west Pico chemiluminescent substrate, Thermo Scientific). Then the blots were exposed to X- ray film (Hyperfilm ECL, GE Healthcare, USA) and developed by using a hyper processor (Model -SRX -101A, Amershan Biosciences). Gel stained with coomassie blue served as an equal loading control. Quantification of band intensity was performed using the Image J Software (NIH, Bethesda, MD, USA). For quantitative analysis, the signal intensity of each of the protein was normalized with the corresponding β-actin signal.

### Statistical analysis

All values were expressed as mean ± SEM. Data were statistically analyzed using student unpaired ‘t’ test for group wise comparison. Significance was set at P≤0.05.

## Results

### Development of hypertrophy in SD rats

**Figure 1:**
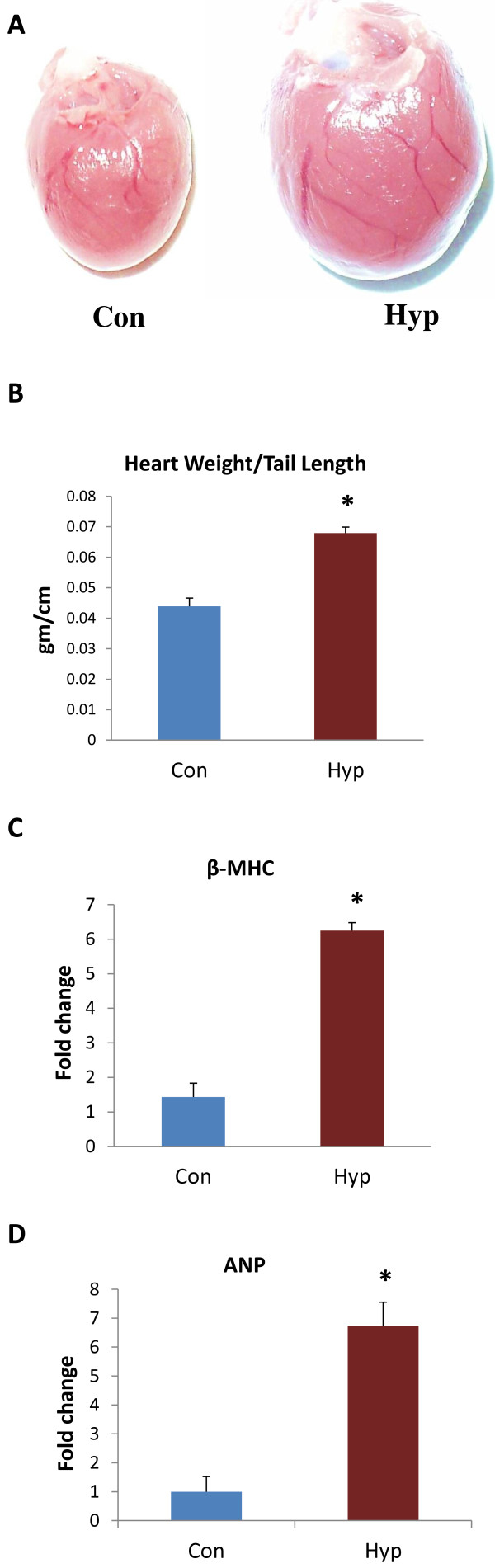
**Chronic administration of isoproterenol caused cardiac hypertrophy in SD rats.** (**A**) Images of whole heart from control and isoproterenol-treated SD rat. (**B**) Heart weight and tail length ratio from control and isoproterenol-treated SD rat. (**C**) β-MHC gene expression from both control and hypertrophic heart measured by Real-time PCR. (**D**) ANP gene expression from both control and hypertrophic heart measured by Real-time PCR. Data shown as mean ± SEM, * p ≤0.05 versus control.

Isoproterenol (5 mg/kg/day) was administered through subcutaneous route for 14 days to induce cardiac hypertrophy. There was 55% increase in heart weight and tail length ratio observed in hypertrophy (Hyp) group compared to control (Con) group (Figure 1A & B). The mRNA levels of hypertrophic marker, β-MHC and ANP were analyzed by Real time PCR which showed elevated levels of this mRNA in Hyp heart compared to Con heart (Figure 1C & D).

### Proteomic assessment of cardiac hypertrophy

**Figure 2:**
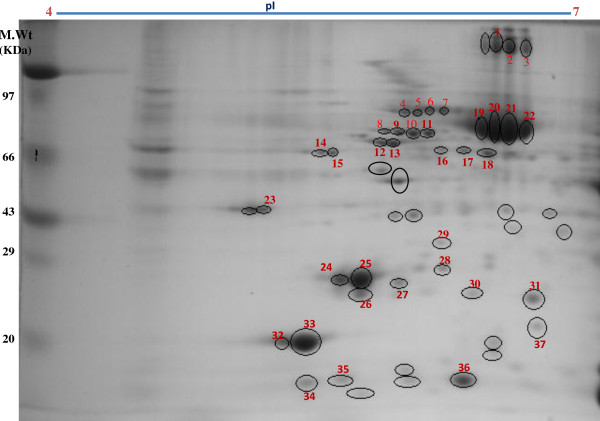
**2-DE gel analysis of proteins extracted from the cardiac tissue of SD rats.** In the first dimension, 200 μg of protein was loaded on a 7 cm IPG strip with a linear gradient of pH 4–7. In the second dimension, 12% SDS –PAGE gels were used. Proteins were visualized by fast coomassie staining. All the differentially expressed spots were marked and identified by MS-analysis. Sizes of the molecular mass markers (in KDa) are indicated.

**Figure 3:**
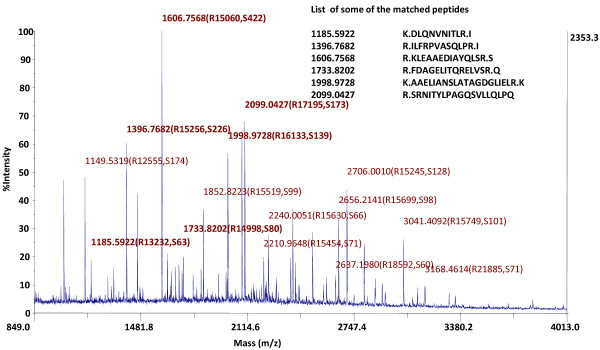
**MALDI-TOF mass spectra of the prohibitin protein isolated from the 2D gels of protein extracts of cardiac tissue.** Some of the matched peptides are labelled bold on the peaks.

Using the above hypertrophy data as guide, we proceeded to examine the global protein complement of left ventricular muscle isolated from control and hypertrophied heart to identify the proteins exhibiting altered abundance as a function of disease progression. For preliminary comparison of the expression levels of total protein extracted from the left ventricle of control and hypertrophy heart samples, we performed one dimensional gel electrophoresis. Additional file 1 (Additional material) shows the representative 1-DE gel image of proteins isolated from cardiac tissue of control and hypertrophic SD rats (Additional file 1: Figure S1). However, since each band in 1D can be a mixture of one, two or more proteins, the results of MALDI-TOF MS analysis indicated protein identification with low MOWSE scores (data not shown). Therefore, to further improve the resolution of separation and identification of differentially expressed proteins in control and hypertrophic samples we carried out two dimensional gel electrophoresis. Proteins (200 μg) extracted and estimated as described in materials and methods for the chosen samples, were separated in the first dimension by isoelectric focusing on strip gel with pI range 3–10 and in the second dimension using 12.0% SDS–PAGE followed by Fast coomassie staining method. 2-DE of the samples on a 3–10 pI IPG strip indicated better resolution of proteins in the pI range of 4–7 and therefore 2DE was carried out using IPG strips with pI 4–7. The protein profile of control and hypertrophic heart was compared. The protein spots which were differentially expressed in both the samples are indicated in Figure 2. These bands were picked up and digested by in-gel trypsin digestion. This was followed by identification with MALDI-TOF MS. The peptides mass peaks were compared with those in the NCBI database (Figure 3).

### The functional cardiac proteome

**Figure 4:**
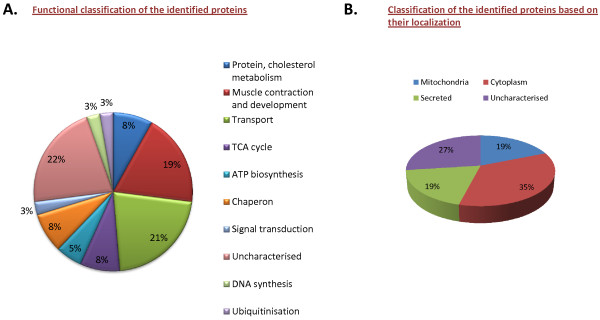
**Classification of identified proteins based on their function and cellular localization.** (**A**) Pie chart grouping the identified proteins in functional classes annotated using expasy database (http://expasy.org/uniprot). (**B**) Pie chart grouping the identified proteins based on their cellular localization.

We initially focused on identifying the proteins of the cardiac proteome from the control sample. The conditions used for profiling 2-D gel allowed access to a total of ~60 visible proteins. All these spots were marked in the control sample and in-gel trypsin digestions followed by mass spectrometry were performed for subsequent identification. Figure 2 shows the representative 2-D gel of proteins isolated from cardiac tissue of control SD rats where the picked and identified protein spots are indicated. A total of 37 proteins were identified (Additional file 2: Figure S2 & Table 2) with significant score. The functional classification of the identified proteins resulted in the following major categories: Cholesterol and protein metabolism, muscle contraction and development, transport, TCA cycle, ATP-biosysnthesis, chaperone, signal transduction, DNA synthesis and ubiquitinisation. Figure 4A shows the pie chart grouping of the identified proteins into functional classes annotated using the expasy database (http://expasy.org/uniprot). Figure 4B shows the pie chart grouping of the identified proteins based on their cellular localization.

**Table 2:** List of proteins identified from 2D gels of protein extracts of cardiac tissue

**Spot no.**	**Category**	**Accession no.**	**Protein name**	**No. of matched peptides**	**Protein score**	**M.Wt**	**pI**
1.	Uncharacterised	gi|55628	Unnamed protein product [rattus norvegicus]	14	75	70670	6.09
2.	Transport	gi|149033753	Albumin, isoform cra_a [rattus norvegicus]	13	78	53060	6.72
3.	Transport	gi|149033753	Albumin, isoform cra_a [rattus norvegicus]	10	87	53060	6.72
4.	Chaperone	gi|1000439 +gi|178847300	grp75 [rattus sp.] chain a, crystal structure of the 70-kda heat shock cognate protein	31	110	73984	5.87
						+59895	5.91
5.	Uncharacterised	gi|149041392	rcg57965, isoform cra_b	9	65	48623	6.16
6.	Signal transduction	gi|149022678	Mitogen activated protein kinase 8 interacting protein, isoform cra_d	6	47	75419	4.73
7.	Chaperone	gi|1000439	grp75 [rattus sp.]	10	72	73984	5.87
8.	Muscle contraction and development	gi|149058891	Calpastatin, isoform cra_a	4	43	20886	5.20
9.	Uncharacterised	gi|1334284	Unnamed protein product	8	47	58061	5.35
10.	Chaperone	gi|56383	Heat shock protein (hsp60) precursor	17	90	61098	5.91
11.	Uncharacterised	gi|1334284,	Unnamed protein product	13	74	58061	5.35
12.	Muscle contraction and development	gi|11968118	Desmin	7	61	53481	5.21
13.	Muscle contraction and development	gi|11968118	Desmin [rattus norvegicus]	29	226	53481	5.21
14.	ATP biosynthesis	gi|1374715	ATP synthase beta subunit	32	159	51171	4.92
15.	ATP biosynthesis	gi|1374715	ATP synthase beta subunit	22	140	51171	4.92
16.	Uncharacterised	gi|293344961	predicted: hypothetical protein	7	45	32379	8.55
17.	TCA cycle	gi|149025181	Dihydrolipoamide s-succinyltransferase (e2 component of 2-oxo-glutarate comp.	7	55	21198	6.92
18.	TCA cycle	gi|149025181	Dihydrolipoamide s-succinyltransferase (e2 component of 2-oxo-glutarate comp.	5	55	21198	6.92
19.	Transport	gi|149033753	Albumin, isoform cra_a	13	87	53060	6.72
20.	Transport	gi|149033753	Albumin, isoform cra_a	14	83	53060	6.72
21.	Transport	gi|149033753	Albumin, isoform cra_a	13	82	53060	6.72
22.	Transport	gi|149033753	Albumin, isoform cra_a	13	78	53060	6.72
23.	Uncharacterised	gi|149066551	rcg59557	5	54	12861	9.18
24.	Uncharacterised	gi|149063941	rcg23467, isoform cra_a	5	51	223560	5.59
25.	Muscle contraction and development	gi|6981240,	Myosin light chain 3	16	97	22256	5.03
26.	Muscle contraction and development	gi|6981240,	Myosin light chain 3	13	55	22256	5.03
27.	Protein, cholesterol metabolism	gi|149063506	Phosphatidylethanolamine binding protein 1, isoform cra_a	7	42	10775	6.26
28.	Protein, cholesterol metabolism, transport	gi|6978515	Apolipoprotein a-i preproprotein	3	43	30100	5.52
29.	DNA synthesis	gi|6679299	Prohibitin	5	141	29859	5.57
30.	Uncharacterised	gi|149054764	rcg33654, isoform cra_d	14	48	16329	5.45
31.	Ubiquitinisation	gi|149016294	F-box only protein 36 (predicted), isoform cra_c	4	40	9895	9.52
32.	Muscle contraction and development	gi|127167	Myosin regulatory light chain 2, ventricular/cardiac muscle isoform	6	120	18868	4.86
33.	Muscle contraction and development	mlrv_rat	mlrv_rat, myosin regulatory light chain 2, ventricular/cardiac muscle isoform	12	169	18868	4.86
34.	Uncharacterised	gi|293349387	Predicted: hcg2036631-like	23	54	51880	9.69
35.	Protein,cholesterol metabolism	gi|149043202	Calpain 9	7	43	51956	5.79
36.	Transport	gi|13162363	Fatty acid-binding protein, heart	9	96	14766	5.91
37.	Muscle contraction and development	gi|20302069	Heat shock protein beta-6	9	68	17551	6.05

### Comparative proteome profiling of control and hypertrophic heart tissues

**Figure 5:**
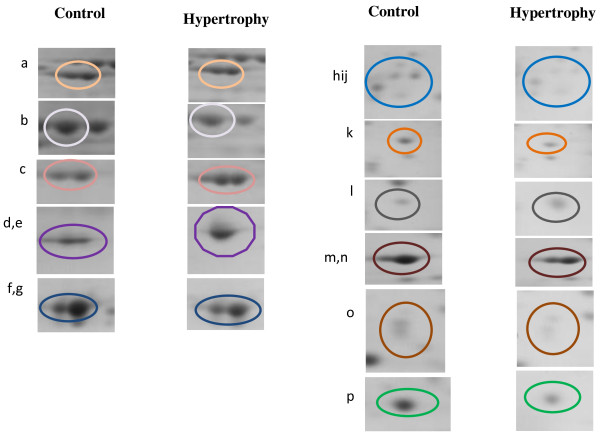
**Close up areas of the gels showing variation in the intensity of the differentially expressed protein spots among the control and the hypertrophic SD rats.** For ease of comparision, spots are indicated by circle. Control is used as a reference for comparision. The identity of the proteins is as follows: **A**. gi|11968118 desmin; **B**. gi|56383 heat shock protein (hsp60) precursor; **C**. gi|1374715 ATP synthase beta subunit; **D,E**. rCG41226; isoform CRA_b [Rattus norvegicus] rCG59557; **F,G**. gi|149063941, rCG23467, isoform CRA_a, gi|6981240, myosin light chain 3; **H,I,J**. gi|149063506, phosphatidylethanolamine binding protein 1, isoform CRA_a, gi|6978515, apolipoprotein A-I preproprotein gi|6679299, prohibitin; **K**. gi|149016294, F-box only protein 36 (predicted), isoform CRA_c; **L**. gi|20302069 Heat shock protein beta-6; **M,N**. gi|127167 Myosin regulatory light chain 2, ventricular/cardiac muscle isoform, gi|127167 Myosin regulatory light chain 2, ventricular/cardiac muscle isoform; **O**. gi|149043202, calpain 9 (nCL-4); **P**. gi|13162363, fatty acid-binding protein, heart.

In an effort to gain insight into the molecular mechanisms underlying the pathogenesis of cardiac hypertrophy, we also performed a comparative analysis of the protein expression profiles of control and cardiac hypertrophy samples of rats by image analysis of 2D gels using ‘progenesis same spots software’. This resulted in the identification of several differentially expressed protein spots between control and cardiac hypertrophy gels. These differentially expressed proteins of cardiac hypertrophy are shown in Figure 5 and listed in Table 2. These include fatty acid binding protein, heart (H-FABP) (spot no. 36), phophatidylethanolamine binding protein (PEBP1) (spot no. 27), apolipoprotein A-1 preprotein (spot no.28), calpain 9 (spot no.35), heat shock cognate protein, 70 kda (HSP70) (spot no.4), heat shock protein 60 precursor (HSP60) (spot no. 10) and heat shock protein B6 (HSPB6) (spot no.37), mitogen activated protein kinase 8 interacting protein (isoform CRA-d) (spot no.6), myosin light chain 3 (MYL3) (spot no.24,25), myosin light chain 2 (MYL2) (spot no. 32,33), desmin (spot no.12,13), dihydrolipoamide succinyltransferase (spot no.18), F-box only protein 36 (isoform CRA_c) (spot no. 31), ATP synthase β-subunit (ATP5β) (spot no.14,15) and prohibitin (PHB) (spot no. 29) (Figure 5).

### Gene expression analysis for the validation of proteomics data

**Figure 6:**
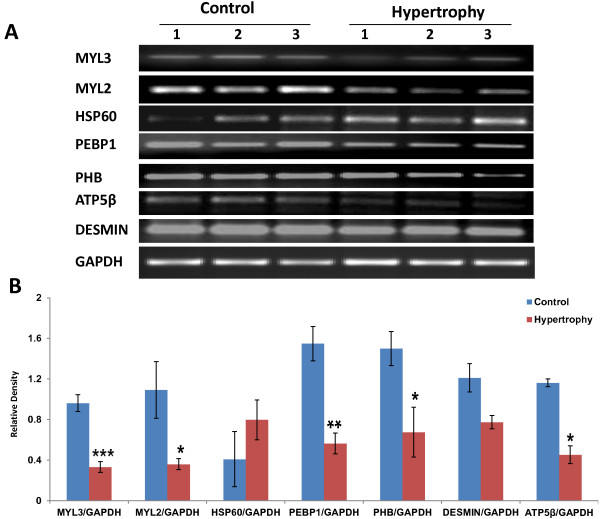
**Gene expression analysis by RTPCR.** (**A**) Gene expression of MYL3, MYL2, HSP60, PEBP1, PHB, ATP5β and Desmin in control and hypertrophy heart. GAPDH is loading control for RTPCR. (**B**) Densitometry analysis (relative density against GAPDH) of all genes obtained by RTPCR. Data shown as mean ± SEM, * p ≤0.05; **P≤0.005; ***P≤0.0005 versus control.

We attempted further validation of seven differentially expressed proteins by semiquantitative RTPCR to check the mRNA expression levels at the transcriptional level (Figure 6). There was a significant (p<0.05) decrease in levels of MYL2, MYL3, PEBP1, PHB and ATP5β gene levels in hypertrophy (Hyp) group compared to control (Con). Although, there was an increase in HSP60 and decrease in Desmin gene expression levels observed in the hypertrophy (Hyp) group compared to Control (Con), these changes were not significant. GAPDH was used as an internal control for normalization in the gene expression studies.

### Immunoblot analysis for the validation of the proteomics data

**Figure 7:**
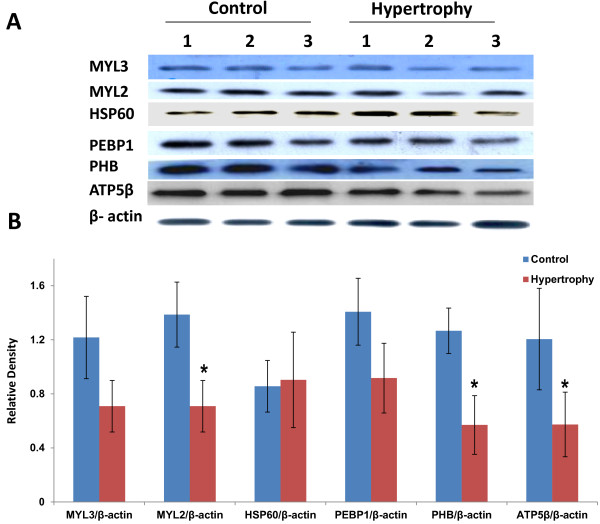
**Protein expression analysis by immunoblots.** (**A**) Protein expression of MYL3, MYL2, HSP60, PEBP1, ATP5β and PHB in control and hypertrophy heart. β- actin is used as a loading control for immunoblot analysis. (**B**) Densitometry analysis (relative density against β- actin) of all proteins obtained by immunoblots. Data shown as mean ± SEM, * p ≤0.05 versus control.

Immunoblot analysis was performed for these differentially expressed proteins with specific antibodies to validate the levels obtained from 2-DE data. β- actin was used as a reference for comparison of protein levels. There was no change in HSP60 expression observed in hypertrophy (Hyp) group compared to control (con). However, significant decrease in protein levels was observed for MYL2, ATP5β and PHB compared to control (Figure 7). Although there was a decrease in protein levels for both MYL3 and PEBP1 in Hyp group compared to Con but it was not significant.

## Discussion

Several attempts have been tried on cardiac tissues to disclose underlying molecular pathogenesis of cardiac hypertrophy in different disease models [14]. However, there is no proteomic study to identify important targets or biomarkers from isoproterenol-induced hypertrophy heart. To the best of our knowledge, this study considerably resolves the proteome of healthy and diseased hearts to investigate and understand the complex mechanism of isoproterenol induced cardiac hypertrophy. Table 2 provides a summary of the list of proteins identified from the 2D gels of cardiac tissue of SD rats. By this approach we were also able to identify several proteins which were specifically and significantly altered in cardiac hypertrophy.

In the present study, we demonstrate that isoproterenol induced cardiac hypertrophy effects at the molecular level, affecting the cardiac protein expression profiles in SD rats. The protein(s) of the following functional categories are altered, the expression of which were further validated by RTPCR and western blotting: (i) Muscle contraction and development, (ii) Transport, (iii) Chaperones, (iv) ATP biosynthesis, (v) DNA synthesis. An intriguing question which rises following this study is whether this differential protein expression analysis might reveal some novel plausible candidates which could act as master regulators responsible for hypertrophy. Identification of novel target is critical to develop new class of drug molecules to modulate or alter the protein function in hypertrophy. Therefore, we initially provide details of proteins which were differentially expressed in our study of isoproterenol-induced hypertrophy and the expression of which are in consistence with the prior studies in other models (such as endothelin-1(ET-1) or leukemic inhibitory factor (LIF) induced hypertrophy models or other models of heart failure, strokes, myocardial infarction MI(s) etc.), followed by detailed description of protein prohibitin, which we speculate as a novel potential biomarker in isoproterenol-induced hypertrophy.

Concerning the category (i), we observed a reduction in MYL2 and MYL3 protein levels in isoproterenol-induced hypertrophy compared to control indicating cardiac damage. Myosin light chain 2 (MYL2), also known as myosin regulatory light chain is implicated in myosin ATPase activity and smooth muscle contraction. Mutations in MYL2 are well associated with familial cardiac hypertrophy [15]. Similarly, MYL3 is associated with cardiac microfibrillar assembly and heart contraction [16]. Changes in the levels of myosin light chain 2 and myosin light chain 3 (MYL2 and MYL3) proteins upon isoproterenol treatment, observed in our study are consistent with the earlier observations of heart failure or cardiomyopathy [17]. Down regulation of MYL2 was observed in human heart failure tissues and this data suggested that MYL2 may play a role in the development and progression of chronic heart failure [18]. Serum proteomic signature of human chagasic patients, (caused by infection with trypanosoma cruzi) where there is a decline in heart function, revealed decrease in MYL 2 levels [19]. Desmin is another protein of category I, which along with other proteins acts as a major stress bearing element in the sarcomere. The levels of this protein are reported to increase along with other cytoskeletal proteins such as tubulin, vinculin and vimentin under conditions of idiopathic dilated cardiomyopathy [20] and also during transition from compensated ventricular hypertrophy to heart failure [21]. However, in contrast to other studies we observed a decrease in desmin gene expression levels in isoproterenol-induced hypertrophy. This might be due to different type of cardiac hypertrophy model and early stages of heart failure.

Category (ii) includes transport proteins such as fatty acid binding protein, Heart (H-FABP), which interestingly showed decrease in the levels in the hypertrophic heart in our 2D gels. H-FABP is known to be involved in fatty acid beta oxidation, intracellular utilization of fatty acids and long chain fatty acid transporter activity. It has been earlier reported as a serum marker for the early diagnosis of stroke as well as acute and chronic myocardial infarctions. H-FABP levels are significantly elevated above their threshold level within 3hrs of myocardial infarction and subsequently return to normal levels within 12-24 hrs [22]. However coronary ligation (MI and ramipril treatment) decreased the protein expression of H-FABP in the left ventricular tissue compared to sham group. In addition, ramipril (known for beneficial role in cardiovascular diseases) treatment caused an increase in H-FABP of rabbit LV with MI indicating that ramipril may modulate the expression of H-FABP by post translational modifications [23].

The Heat shock proteins (HSP60, HSP70 and HSPD1), representing category (iii) increased in the isoproterenol-induced disease model in comparison to control in the present study. Interestingly, these proteins are known to change in abundance during cardiac hypertrophy *in vitro* and have also been shown to change in human/animal models of hypertrophy [24]. Proteomic analysis revealed significant elevation of heat shock protein 70 in patients with chronic heart failure due to arrhythmogenic right ventricular cardiomyopathy (ARVC). Results of the study indicated that elevated HSP70 levels as the common feature of heart failure due to ARVC, DCM, and ICM suggesting that HSP70 may be used as a biomarker for the presence of heart failure due to cardiomyopathies of different etiologies and may hold diagnostic/prognostic potential in clinical practice [25]. Also, proteomic analysis in congestive heart failure revealed significant alternations of cardiac small heat shock protein expression [26].

Concerning the effect of Isoproterenol on ATP biosynthesis (category IV), the energetics of hypertrophy (LV) and heart failure have been extensively studied. In heart failure, abnormalities are found in myocardium relating to substrate use, oxidative phosphorylation, and the high energy transfer mechanism involving creatine. Consisent with this we observed a decrease in ATP-synthase 5β levels in hypertrophic heart compared to control heart. However, cardioprotection against hypobaric hypoxia showed elevated levels of ATP-synthase 5β suggesting improvement in mitochondrial energy metabolism [27].

In the present study, we identified prohibitin, a mitochondrial protein, as differentially expressed and decreased significantly in hypertrophic heart compared to control (Figure 5 and Figure 6). Prohibitins are ubiquitous, evolutionarily conserved proteins that are mainly localized in mitochondria [28]. Although the accumulating evidence suggests that prohibitins are localised primarily within mitochondria, their function remains poorly understood. Studies in different organisms have provided significant insights into the role of the prohibitin complex in mitochondrial biogenesis and metabolism [29]. Mitochondrial dysfunction is believed to be involved in the process of free radical generation, leading to hypertrophy and heart failure [30]. Isoproterenol-induced cardiac injury is associated with mitochondrial disorder affecting one or more of the respiratory redox chain [31]. The heart tissues, which rely most extensively on aerobic metabolism, are most severely affected by mitochondrial defects. Therefore, attention has been recently focused towards investigation of mitochondrial function and its biogenesis.

To our knowledge, this study provides the first experimental evidence for decreasing levels of prohibitin (PHB) expression in hypertrophic rat heart. The expression of PHB1 is also down-regulated after induction of oxidative stress in epithelial cells as well as in diseases linked to enhanced reactive oxygen species (ROS) such as ulcerative colitis and Crohn’s disease [32] Oxidative stress has been identified as one of the key contributing factors in the progression and development of cardiac hypertrophy. Several research articles support the fact that ROS generated from mitochondria cause cardiac hypertrophy [9]. Although increased ROS and mitochondrial dysfunction are associated with cardiac hypertrophy, it is not known if PHB is able to collectively control these events or have an impact on cardiac hypertrophy. Knockdown of prohibitin (PHB1) in endothelial cells increases mitochondrial production of reactive oxygen species via inhibition of complex I [33]. It is possible that decreased expression of PHB in hypertrophy heart might be responsible for mitochondrial dysfunction and oxidative stress. Evidence of increased oxidative stress in isoproterenol-induced cardiac hypertrophy was reported earlier [34].

Therefore, we hypothesize that the reduced expression of PHB might be responsible for mitochondrial dysfunction and increased oxidative stress in isoproterenol-induced cardiac hypertrophy. Future treatment strategy can be initiated to increase mitochondrial PHB by pharmacological and/or therapeutic agents. Alternatively, PHB can also be used as a potential biomarker for stress induced cardiac hypertrophy.

## Conclusions

The successful use of multiple techniques, including 2-DE and MALDI-TOF-MS demonstrates that proteomic analysis provides appropriate means for identifying cardiac biomarkers in hypertrophy. The analysis of changes in myocardial protein expression may serve as source of possible biomarkers for hypertrophic cardiac damage. Our data offer new insights into understanding the cellular and molecular mechanisms that underlie cardiac dysfunction in cardiac hypertrophy induced by “a catecholamine”. The present study identifies alteration in the prohibitin levels, as a key actor and contributor to the progression of cardiac hypertrophy and heart failure. Data obtained in experimental animals must be extrapolated to the clinical arena for further investigation. Nevertheless, the findings from the present study show that prohibitin could be useful to predict the therapeutic response to established and novel therapies for the prophylaxis of cardiac hypertrophy and subsequent heart failure in cardiac patients.

## Abbreviations

2DE: 2 Dimensional Gel Electrophoresis; SD: Sprague Dawley; PMF: Peptide mass fingerprinting; MS: Mass Spectrometry; MALDI: Matrix-assisted laser desorption/ionization; TOF: Time of flight.

## Competing interests

The authors declare that they have no conflict of interests.

## Authors’ contributions

DC and TAD designed and carried out most of the experiments, data analysis and analyzed the proteomic data. TNK developed and confirmed the animal models of hypertrophy; PS did the MS analysis of samples and provided the mass spectrometry data. SKB guided the development of animal models. SKB and MPB conceived the study, participated in designing and coordinated in drafting the manuscript. All authors read and approved the final manuscript.

## Supplementary Material

Additional file 1: Figure S1 1-D 10% SDS-PAGE of control and hypertrophic heart. Approx 20 μg of protein from control (C1, C2, C3) and hypertrophic samples (H1, H2, H3) was separated on SDS PAGE and analyzed for identification of differentially expressed protein(s). Click here for file

Additional file 2: Figure S2 Protein profiling of the control and hypertrophic heart by two dimensional (2D) gel electrophoresis. Proteins were separated on 2D PAGE and visualized by fast coomassie staining. Labelled spots correspond to differential proteins analyzed and identified by MS-analysis. Click here for file
