# Supplementary figures and images for: A proteomic view of isoproterenol induced cardiac hypertrophy: Prohibitin identified as a potential biomarker in rats
Source: J Transl Med. 2013 May 24;11:130. doi: 10.1186/1479-5876-11-130 (PMC3667141; doi:10.1186/1479-5876-11-130)

## Slide 1
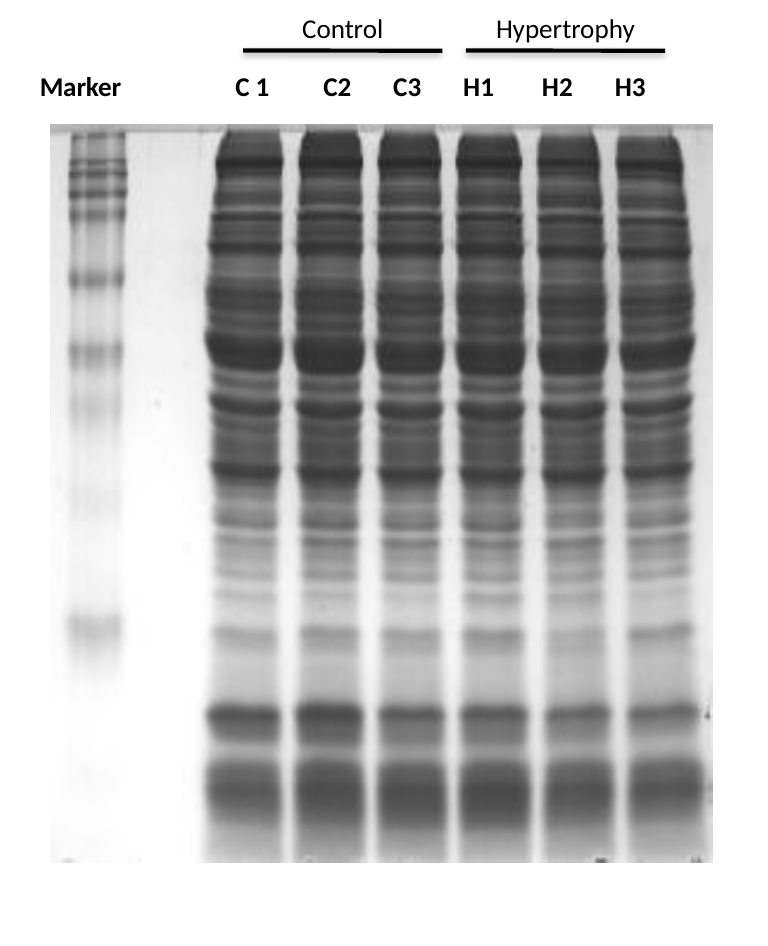

Control
Hypertrophy
Marker C 1 C2 C3 H1 H2 H3

Supplement: Additional file 1: Figure S1 — 1-D 10% SDS-PAGE of control and hypertrophic heart. Approx 20 μg of protein from control (C1, C2, C3) and hypertrophic samples (H1, H2, H3) was separated on SDS PAGE and analyzed for identification of differentially expressed protein(s). [file 1479-5876-11-130-S1.pptx]

## Slide 1
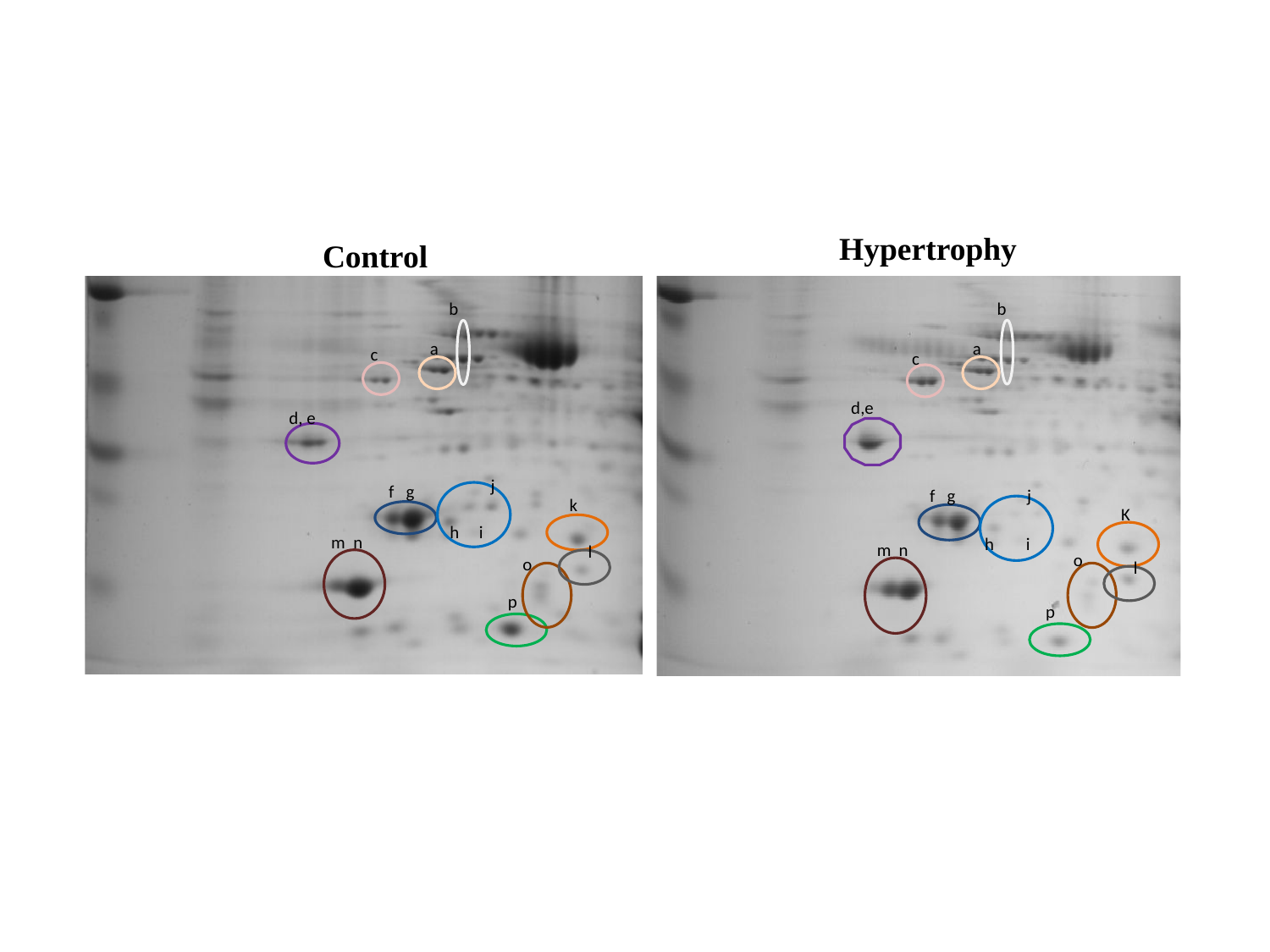

Hypertrophy
b
a
c
d,e
 j
f g
K
h i
m n
o
l
p
Control
b
a
c
d, e
 j
f g
k
h i
m n
l
o
p

Supplement: Additional file 2: Figure S2 — Protein profiling of the control and hypertrophic heart by two dimensional (2D) gel electrophoresis. Proteins were separated on 2D PAGE and visualized by fast coomassie staining. Labelled spots correspond to differential proteins analyzed and identified by MS-analysis. [file 1479-5876-11-130-S2.pptx]
